# Cytotoxic and Synergistic Effects of Environmentally Relevant Binary Pollutant Mixtures in a Human Lymphoblast Cell Line

**DOI:** 10.3390/jox16020039

**Published:** 2026-02-24

**Authors:** Francisco Alejandro Lagunas-Rangel

**Affiliations:** Department of Genetics and Molecular Biology, Centro de Investigación y de Estudios Avanzados del Instituto Politécnico Nacional, Av. Instituto Politécnico Nacional 2508, San Pedro Zacatenco, Gustavo A. Madero, Mexico City 07360, Mexico; francisco.lagunas@cinvestav.mx

**Keywords:** viability, serum/plasma concentration, plasticizers, pollutant mixture

## Abstract

Environmental pollutants are persistent chemicals that pose substantial risks to human health, contributing to global mortality and economic burden. In real-world situations, exposure rarely occurs to single compounds; instead, people are chronically exposed to complex mixtures at low concentrations. However, most regulatory frameworks still rely on single-substance risk assessments, potentially underestimating the hazards associated with combined exposures. This study investigated the cytotoxic interactions of binary mixtures of five environmentally relevant pollutants: bisphenol A (BPA), bisphenol A diglycidyl ether (BADGE), dibutyl phthalate (DBP), di(2-ethylhexyl) phthalate (DEHP), and perfluorooctanoic acid (PFOA), using the human lymphoblast cell line NALM-6. Cells were exposed for 72 h to each compound individually and to all possible binary combinations, reflecting concentrations reported in human plasma or serum. Cell viability was assessed using the 3-(4,5-dimethylthiazol-2-yl)-2,5-diphenyltetrazolium bromide (MTT) assay, and interactions were analyzed using the Bliss model of independence and two-way analysis of variance (ANOVA). Intracellular reactive oxygen species were measured using the 2′,7′-dichlorodihydrofluorescein diacetate (DCFH-DA) probe to explore the involvement of oxidative stress. Synergistic interactions were observed under specific conditions, although not all statistically identified interactions corresponded to biologically significant effects. The BPA-DBP combination produced the highest cytotoxicity when both pollutants were present at 100 nM (31%), consistent with a strong synergistic effect. A similar pattern was observed for BADGE-BPA. ROS production was partially associated with cytotoxicity in these selected mixtures. Overall, these findings highlight the importance of distinguishing statistical synergy from toxicological relevance.

## 1. Introduction

Environmental pollutants are chemical substances released into natural ecosystems that pose serious risks to human health and other living organisms. Many of these compounds are widely distributed and persist in the environment for long periods due to their resistance to degradation, resulting in continuous, low-level exposure in daily life [1,2]. The global impact of pollution on health is substantial. Exposure to environmental pollutants is estimated to cause approximately 9 million deaths annually, accounting for 16% of all deaths worldwide. In economic terms, pollution was responsible for global losses of approximately US$4.6 trillion in 2015, representing 6.2% of global gross domestic product [3]. Despite these profound consequences, pollution has historically received limited attention on international development and global health agendas. As a result, the health burden associated with environmental pollutants remains underrepresented in global disease burden assessments, leading to a systematic underestimation of their true impact [4]. Furthermore, current regulatory frameworks often fail to adequately address the health risks associated with combined exposure to multiple pollutants [5].

In real-world situations, environmental pollutants rarely occur in isolation. Instead, they coexist as complex mixtures [6]. These mixtures can originate from a single source releasing multiple pollutants simultaneously or from different sources whose emissions converge in the same environmental compartments [7]. Within these mixtures, individual pollutants can interact with each other in diverse and often unpredictable ways. These interactions can amplify toxic effects through synergistic mechanisms, diminish them through antagonism, or simply generate additive responses [8,9]. Consequently, the overall biological impact of a pollutant mixture can differ substantially from that predicted based on the effects of each compound separately [10].

Because pollutants can interact synergistically, mixtures of pollutants can cause harmful health effects, even when each individual compound is present at concentrations considered safe on its own. This calls into question the adequacy of current environmental quality standards and regulatory frameworks, which are based primarily on single-compound risk assessments and may therefore underestimate the true risks [11,12]. Consequently, there is a clear need for further research focused on understanding the toxicity of mixtures and incorporating these interactions into more protective regulatory approaches. With these considerations in mind, the aim of this study was to evaluate the interactions of binary mixtures of five common environmental pollutants: bisphenol A (BPA), bisphenol A diglycidyl ether (BADGE), dibutyl phthalate (DBP), di(2-ethylhexyl) phthalate (DEHP), and perfluorooctanoic acid (PFOA), using a human lymphoblast cell line. The study focused on assessing the cytotoxic effects of these binary mixtures at concentrations comparable to those reported in human plasma and serum. To reflect real-world exposure scenarios, concentrations of 1 nM, 10 nM, and 100 nM were tested to identify potential synergistic effects on cell viability. Furthermore, comprehensive statistical analyses were applied to characterize interaction patterns and determine the relative contribution of individual compounds and their combined effects to the observed cytotoxic responses. To try to elucidate the mechanisms underlying cytotoxicity, intracellular reactive oxygen species (ROS) levels were measured in selected combinations where pronounced cytotoxic effects and synergistic interactions were observed.

## 2. Materials and Methods

### 2.1. Pollutants

Stock solutions of the following pollutants were prepared: BPA (Sigma-Aldrich, 133027, Burlington, MA, USA), BADGE (Sigma-Aldrich, D3415, Burlington, MA, USA), DBP (Sigma-Aldrich, 524980, Burlington, MA, USA), DEHP (Sigma-Aldrich, 36735, Burlington, MA, USA), and PFOA (Sigma-Aldrich, 171468, Burlington, MA, USA). Precisely weighed amounts of each compound were dissolved in dimethyl sulfoxide (DMSO; Invitrogen, D12345, Carlsbad, CA, USA) to obtain stock solutions at a final concentration of 10 mM. Working solutions at the concentrations required for each experimental condition were prepared by diluting these stock solutions.

### 2.2. Cell Line and Culture Conditions

The NALM-6 cell line used in this study was obtained from the German Collection of Microorganisms and Cell Cultures GmbH (DSMZ; ACC 128, Braunschweig, Germany). Cells were maintained in RPMI 1640 medium (Sigma, R0883, St. Louis, MO, USA) supplemented with 10% heat-inactivated fetal bovine serum (Sigma, F9665, St. Louis, MO, USA) and 1% penicillin–streptomycin solution (Sigma, P0781, St. Louis, MO, USA). Cultures were incubated at 37 °C in a humidified atmosphere with 5% CO_2_.

### 2.3. Cell Viability Assays

A 3 × 3 factorial experimental design was used to evaluate binary combinations of pollutants at three concentration levels (1 nM, 10 nM and 100 nM). Within this framework, individual pollutants at each concentration were also included as individual design factors. For each experimental condition, at least three independent replicates were performed. Monocultures were established by manually seeding 10,000 cells per well in 100 μL of culture medium in 96-well plates (Corning Falcon, CLS351172, Corning, New York, NY, USA). After a 24 h preculture, cells were treated with the pollutants or corresponding control vehicles, according to a predefined plate arrangement. The final vehicle concentration depended on the pollutant dilution and was 0.001% for 100 nM, 0.0001% for 10 nM, and 0.00001% for 1 nM. Appropriate vehicle controls were included for each concentration in each experiment, and no significant effects on cell viability were observed. After treatment, the plates were incubated for 72 h at 37 °C in a humidified atmosphere containing 5% CO_2_. The culture medium was replaced every 24 h with fresh medium containing the respective pollutants or vehicle control. Cell viability was then assessed using the 3-(4,5-dimethylthiazol-2-yl)-2,5-diphenyltetrazolium bromide (MTT) assay. Briefly, MTT reagent (Invitrogen, M6494, Carlsbad, CA, USA) was added to each well, and the plates were incubated for 1 h to allow metabolically active cells to reduce the tetrazolium salt to insoluble formazan crystals. After incubation, the crystals were solubilized in isopropanol (Sigma-Aldrich, 190764, Burlington, MA, USA), and the absorbance was measured spectrophotometrically. Viability was assessed using a microplate reader (Tecan Sunrise, Tecan Group Ltd., Männedorf, Switzerland). Cell viability was expressed as a percentage relative to the corresponding vehicle control.

### 2.4. Interaction and Synergy Analysis

Interactions between pollutants were assessed using cell viability data analyzed with the SynergyFinder 3.0 platform [13], applying predefined parameters. Synergistic, additive, or antagonistic effects were determined using the Bliss model of independence. In parallel, interactions were statistically evaluated using a two-way analysis of variance (ANOVA), followed by Tukey’s post hoc test for multiple comparisons. All statistical analyses were performed using GraphPad Prism version 9 (GraphPad Software, La Jolla, CA, USA). Data normality was assessed using the Shapiro–Wilk test, and results with *p* ≤ 0.05 were considered statistically significant.

### 2.5. Measurement of Intracellular ROS

Intracellular ROS levels were quantified using the 2′,7′-dichlorodihydrofluorescein diacetate (DCFH-DA) probe (Sigma-Aldrich, 35845-5G, Burlington, MA, USA). Cells were seeded at a density of 10,000 cells per well in 100 μL of culture medium in 96-well black plates with a transparent bottom (Thermo Scientific, 265301, Waltham, MA, USA). After a 24 h preculture, cells were treated with the specified pollutants or corresponding control vehicles, according to a predefined plate arrangement. The final vehicle concentration depended on the pollutant dilution and was 0.001% for 100 nM, 0.0001% for 10 nM, and 0.00001% for 1 nM. After 72 h of treatment, cells were washed once with phosphate-buffered saline (PBS) and incubated with 10 µM of DCFH-DA diluted in serum- and phenol red-free medium (Gibco, 11835030, Grand Island, NY, USA) for 30 min at 37 °C in the dark. Cells were then washed twice with PBS, and 30 µL of PBS was added to each well prior to measurement. Fluorescence was measured using a Victor X3 2030-0030 microplate reader (PerkinElmer, Shelton, CT, USA) with excitation at 485 nm and emission at 530 nm. Background fluorescence from wells containing the probe without cells was subtracted from all readings. Each condition was evaluated in at least three independent experiments. ROS levels were calculated as change relative to the corresponding vehicle control, which was set to 1. The relationship between intracellular ROS levels and cytotoxicity was assessed using Spearman’s rank correlation coefficient (r). Normality of the data was assessed using the Shapiro–Wilk test. As the data did not follow a normal distribution, non-parametric tests were applied. Statistical significance was defined as *p* ≤ 0.05. All analyses were performed using GraphPad Prism version 9 (GraphPad Software, La Jolla, CA, USA).

## 3. Results

### 3.1. BADGE-BPA

**Figure 1:**
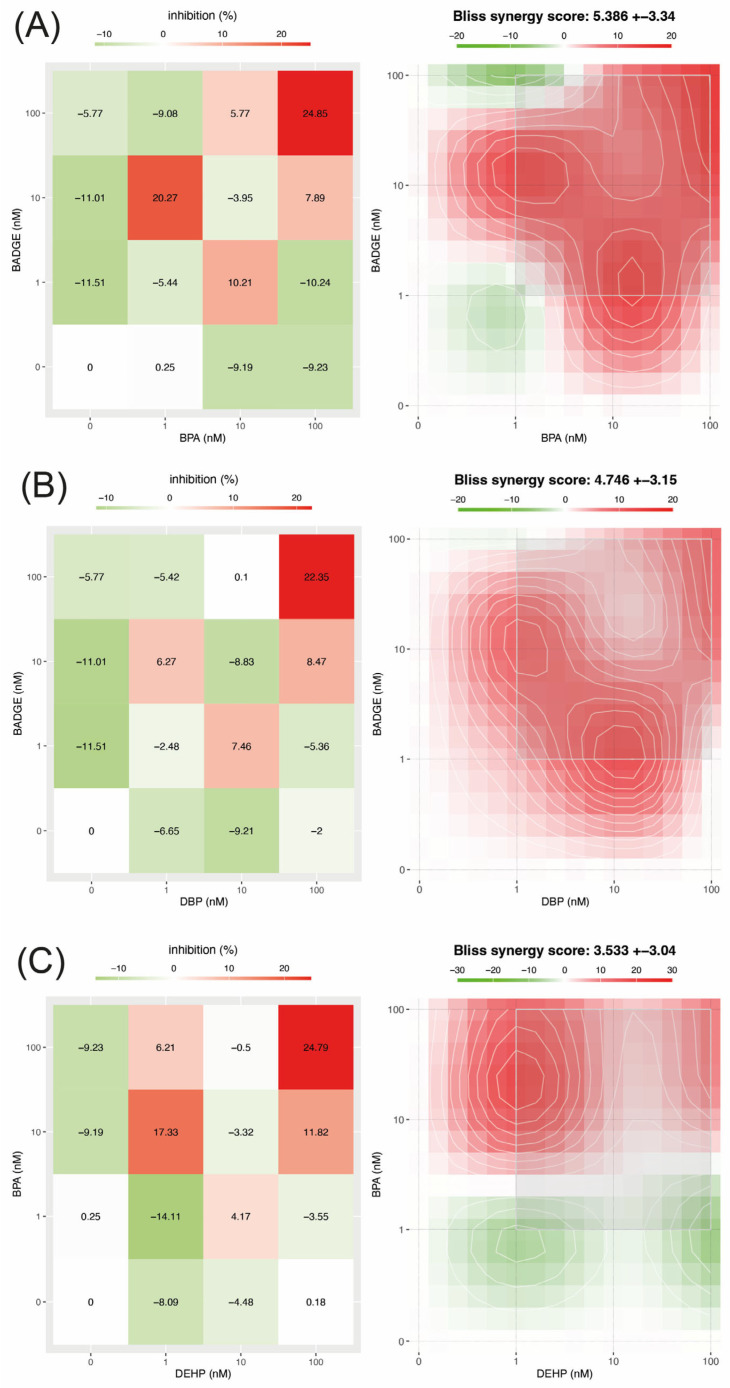
Heatmaps illustrate the cytotoxic and interactive effects of the pollutant combinations BPA-BADGE (**A**), BADGE-DBP (**B**), and BPA-DEHP (**C**). The panels on the left represent the effects on cell viability: red indicates cytotoxicity and green, increased viability; color intensity reflects the magnitude of the effect, and white indicates no change in viability. The panels on the right represent the interaction outcomes: red indicates synergistic effects, green antagonism, and white additive interactions. Gray squares highlight the concentration ranges exhibiting the most intense synergistic interactions for each pollutant combination. In the heatmaps, the first column represents the individual pollutant shown on the *Y*-axis, while the last row represents the individual pollutant shown on the *X*-axis. In both cases, these positions correspond to single-compound conditions at 0, 1, 10, and 100 nM.

Overall, the combination of BPA and BADGE had little effect on cell viability at most of the concentrations tested. However, a marked reduction in viability (greater than 20%) was observed when both compounds were applied at their highest concentration (100 nM), as well as when BPA was combined at a low dose (1 nM) with an intermediate concentration of BADGE (10 nM) (Figure 1A).

Despite the generally modest cytotoxicity, interaction analysis revealed a strong synergistic effect between BPA and BADGE. The mean synergy score for this combination was 5.39 ± 3.34, which was the highest among all the pollutant mixtures evaluated, with a maximum local synergy score of 6.44. The strongest synergistic interactions were detected in combinations of specific concentrations, particularly 1 nM of BPA with 10 nM of BADGE, and in the condition where both compounds were present at the highest concentration tested (100 nM). The combination of 10 nM BPA with 1 nM BADGE also produced a high synergy score, but the associated reduction in cell viability was more moderate (approximately 10%). These findings suggest a partial connection between the calculated synergy scores and the magnitude of the biological effect observed for this combination of pollutants, although this relationship was not uniform across all analyzed conditions. The two-way ANOVA showed that the interaction between BPA and BADGE was the main factor influencing cell viability, explaining 76.99% of the total observed variance (*p* < 0.0001, F = 39.18, DFn = 4, DFd = 18). This indicates that the effect on cell viability depends largely on the specific combination of BPA and BADGE concentrations, rather than on each compound separately. The individual (main) effect of BADGE explained 11.21% of the variance and was statistically significant (*p* = 0.0006, F = 11.41, DFn = 2, DFd = 18). In contrast, the main effect of BPA explained only 2.96% of the variance and was not statistically significant (*p* = 0.0745, F = 3.01, DFn = 2, DFd = 18).

### 3.2. BADGE-DBP

The BADGE-DBP mixture produced minimal changes in cell viability at most concentration combinations. A substantial decrease in viability (greater than 20%) was only detected when both compounds were combined at their highest concentration (100 nM) (Figure 1B). While overall cytotoxic effects were limited, interaction analysis identified synergistic behavior between BADGE and DBP. The average synergy index for this mixture was 4.75 ± 3.15, with a maximum local synergy index of 5.27, indicating that the strongest synergistic interaction occurred with the highest concentration combination (100 nM BADGE and 100 nM DBP). This observation indicates that, at a concentration of 100 nM for both pollutants, the high synergy index was accompanied by a marked cytotoxic effect, suggesting a relationship between the predicted interaction and the biological response at this specific point. However, this pattern was not consistently observed at other concentration combinations, where the synergy indices and cytotoxicity were not related to the same extent. Consistent with these findings, the two-way ANOVA revealed that the interaction between BADGE and DBP was the main contributor to changes in cell viability, explaining 70.68% of the total variance (*p* < 0.0001, F = 28.92, DFn = 4, DFd = 18). The main effect of BADGE alone explained 12.06% of the variance and was statistically significant (*p* = 0.0013, F = 9.87, DFn = 2, DFd = 18), while the main effect of DBP explained a smaller proportion of the variance (6.25%) and was also statistically significant (*p* = 0.0174, F = 5.12, DFn = 2, DFd = 18).

### 3.3. DEHP-BPA

In the case of the DEHP-BPA mixture, notable reductions in cell viability (greater than 20%) were limited to specific concentration pairs, namely, when both compounds were present at their highest concentration (100 nM) and when a low dose of DEHP (1 nM) was combined with an intermediate level of BPA (10 nM) (Figure 1C). These same concentration combinations also corresponded to the strongest synergistic interactions. These findings indicate that, in these specific concentration combinations, higher calculated synergy scores were associated with a greater magnitude of the observed biological effect. However, this pattern was not evident in other concentration combinations, suggesting that the relationship between synergy scores and biological response depended on the specific doses evaluated. The mean synergy index for the DEHP and BPA mixture was 3.53 ± 3.04, with a maximum local synergy index of 4.29. Statistical analysis further supported the importance of the combined exposure effects. Two-way ANOVA indicated that the interaction between DEHP and BPA was the largest contributor to the variability in cell viability, accounting for 48.18% of the total variance (*p* < 0.0001, F = 33.68, DFn = 4, DFd = 18). Furthermore, BPA alone exerted a substantial and statistically significant effect, explaining 30.77% of the variance (*p* < 0.0001, F = 43.02, DFn = 2, DFd = 18), while DEHP independently contributed 14.62% of the variance and was also significant (*p* < 0.0001, F = 20.44, DFn = 2, DFd = 18).

### 3.4. DBP-BPA

**Figure 2:**
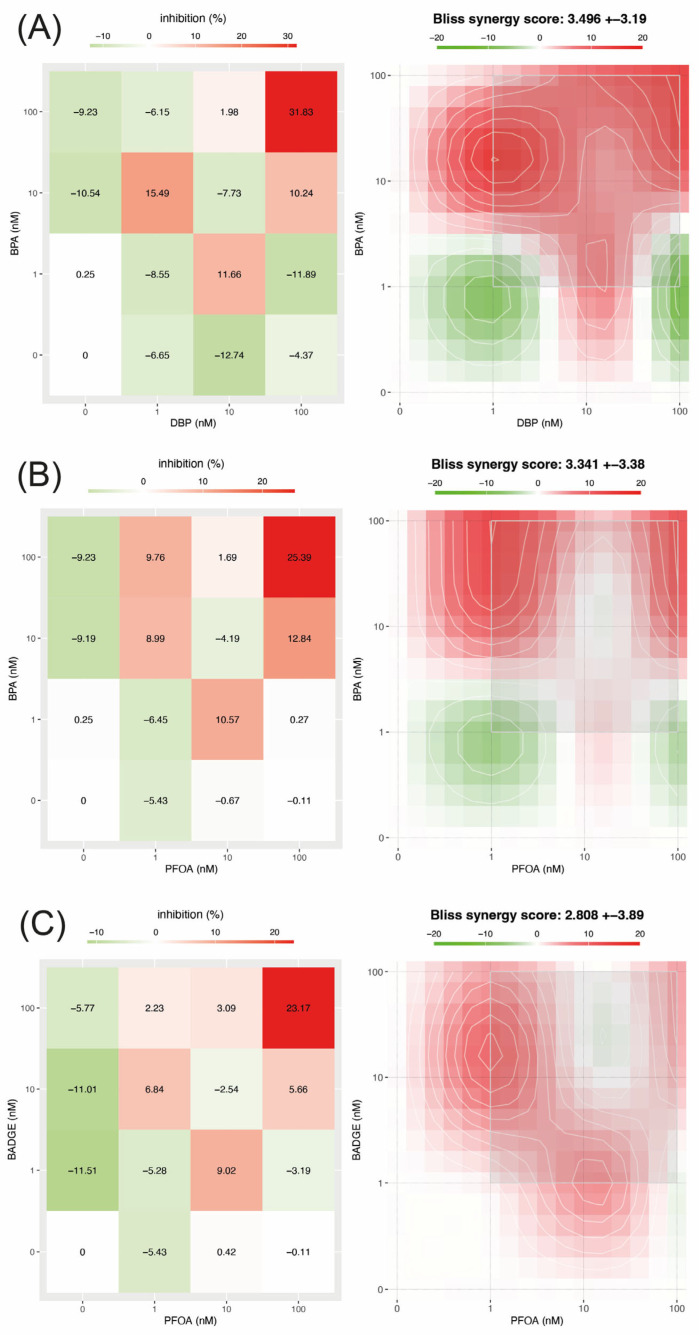
Heatmaps illustrate the cytotoxic and interactive effects of the pollutant combinations BPA-DBP (**A**), BPA-PFOA (**B**), and BADGE-PFOA (**C**). The panels on the left represent the effects on cell viability: red indicates cytotoxicity and green, increased viability; color intensity reflects the magnitude of the effect, and white indicates no change in viability. The panels on the right represent the interaction outcomes: red indicates synergistic effects, green antagonism, and white additive interactions. Gray squares highlight the concentration ranges exhibiting the most intense synergistic interactions for each pollutant combination. In the heatmaps, the first column represents the individual pollutant shown on the *Y*-axis, while the last row represents the individual pollutant shown on the *X*-axis. In both cases, these positions correspond to single-compound conditions at 0, 1, 10, and 100 nM.

For the combination of DBP and BPA, substantial losses in cell viability (greater than 20%) were observed only when both compounds were applied at the highest concentration analyzed (100 nM) (Figure 2A). This combination resulted in the highest level of cytotoxicity observed in the study, with a 31.8% reduction in cell viability. This condition also generated the most pronounced synergistic response, indicating a strong interaction between DBP and BPA at high exposure levels. A weaker, but still relevant, synergistic effect was observed when BPA at 10 nM was combined with DBP at 1 nM, suggesting that interaction effects could also occur at lower concentrations. This represents a clear example where the calculated synergy score is not proportionally reflected in the magnitude of the observed cytotoxic effect, highlighting a discrepancy between the statistical interaction metric and the biological response. Synergy analysis revealed a mean synergy index of 3.496 ± 3.19 for the DBP-BPA mixture, with a maximum local synergy value of 4.54. Two-way ANOVA showed that the interaction between DBP and BPA was the main factor influencing cell viability, explaining 71.16% of the total variance (*p* < 0.0001, F = 79.95, DFn = 4, DFd = 18). In contrast, the individual effects of BPA and DBP explained much smaller, though still significant, proportions of the variance (11.89% [*p* < 0.0001, F = 26.73, DFn = 2, DFd = 18] and 12.94% [*p* < 0.0001, F = 29.06, DFn = 2, DFd = 18], respectively).

### 3.5. PFOA-BPA

Similar to other combinations, the PFOA–BPA mixture produced a pronounced reduction in cell viability exceeding 20% only when both compounds were applied simultaneously at the highest concentration tested (100 nM) (Figure 2B). This condition also corresponded to the strongest synergistic response. In other combinations of concentrations, no consistent relationship was observed between the magnitude of cytotoxicity and the calculated synergy scores. Although the overall cytotoxicity of this mixture was moderate, interaction analysis showed that PFOA and BPA did not act independently. Synergy assessment yielded a mean synergy score of 3.341 ± 3.38, with a maximum local score of 4.3, supporting the presence of interaction-driven effects beyond simple additivity. Two-way ANOVA further emphasized the importance of the combined exposure, revealing that the BPA-PFOA interaction was the main determinant of changes in cell viability, accounting for 46.19% of the total variance (*p* < 0.0001, F = 13.49, DFn = 4, DFd = 18). When analyzed separately, both compounds also had significant, albeit smaller, effects. BPA alone explained 17.97% of the variance (*p* = 0.001, F = 10.50, DFn = 2, DFd = 18), while PFOA accounted for 20.43% (*p* = 0.0005, F = 11.94, DFn = 2, DFd = 18).

### 3.6. BADGE-PFOA

The combination of BADGE and PFOA resulted in a clear reduction in cell viability of more than 20% only when both compounds were present at the highest concentration analyzed (100 nM) (Figure 2C).

This exposure scenario also generated the most intense synergistic response, indicating that the combined action of these compounds is most significant at higher concentrations. Although the additional conditions showed moderate synergy scores, the corresponding cytotoxic effects were minimal, generally below 6%, indicating limited biological relevance despite the statistical interaction. While the overall cytotoxic effect of the mixture was limited, interaction analysis showed that the observed response could not be explained by the effects of either BADGE or PFOA alone. Synergy analysis supported this interpretation, yielding a mean synergy score of 2.808 ± 3.89 and a maximum local score of 3.08, consistent with interaction-driven effects outweighing simple additivity. Statistical evaluation using two-way ANOVA further highlighted the predominance of the combined exposure, with the BADGE-PFOA interaction accounting for nearly half of the total variability in cell viability (48.47%, *p* < 0.0001, F = 14.83, DFn = 4, DFd = 18). When evaluated independently, BADGE contributed most strongly to the observed effects, explaining 25.02% of the variance (*p* = 0.0001, F = 15.31, DFn = 2, DFd = 18), while PFOA accounted for a smaller but still significant proportion (11.80%, *p* = 0.0050, F = 7.23, DFn = 2, DFd = 18).

### 3.7. PFOA-DBP

**Figure 3:**
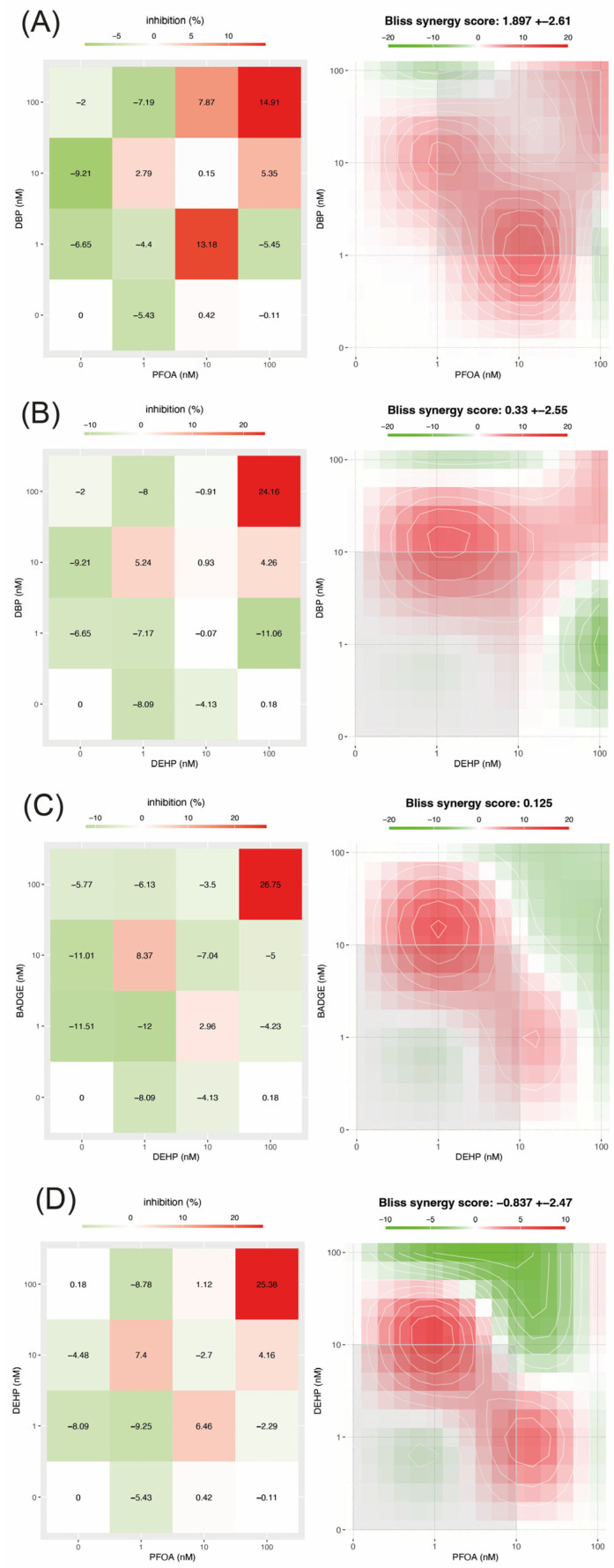
Heatmaps illustrate the cytotoxic and interactive effects of the pollutant combinations DBP-PFOA (**A**), DBP-DEPH (**B**), BADGE-DEHP (**C**), and DEHP-PFOA (**D**). The panels on the left represent the effects on cell viability: red indicates cytotoxicity and green, increased viability; color intensity reflects the magnitude of the effect, and white indicates no change in viability. The panels on the right represent the interaction outcomes: red indicates synergistic effects, green antagonism, and white additive interactions. Gray squares highlight the concentration ranges exhibiting the most intense synergistic interactions for each pollutant combination. In the heatmaps, the first column represents the individual pollutant shown on the *Y*-axis, while the last row represents the individual pollutant shown on the *X*-axis. In both cases, these positions correspond to single-compound conditions at 0, 1, 10, and 100 nM.

In the case of the PFOA and DBP mixture, cell viability decreases greater than 10% were observed only under specific exposure conditions: when both compounds were applied at the highest concentration analyzed (100 nM) and when 10 nM of PFOA was combined with 1 nM of DBP (Figure 3A). These two conditions also coincided with the most intense synergistic interactions, indicating that the combined effects occur only at specific concentrations, and not across the entire exposure range. Consistent with this observation, synergy analysis showed relatively modest but measurable interaction effects, with a mean synergy index of 1.897 ± 2.61 and a maximum synergistic area score of 2.25. Synergistic interactions were detected in this combination of pollutants. However, these did not result in measurable increases in cytotoxicity, suggesting limited biological relevance. While the overall cytotoxicity of this mixture was limited, statistical analysis highlighted the importance of interactions between the compounds. Two-way ANOVA revealed that the interaction between PFOA and DBP was the dominant factor affecting cell viability, explaining 56.33% of the total variance (*p* < 0.0001, F = 46.73, DFn = 4, DFd = 18). When considering individual effects, PFOA had a substantially greater contribution than DBP. The main effect of PFOA accounted for 33.97% of the variance and was highly significant (*p* < 0.0001, F = 56.36, DFn = 2, DFd = 18), whereas DBP alone explained only 4.28% of the variance, although this effect was also statistically significant (*p* < 0.0001, F = 7.10, DFn = 2, DFd = 18).

### 3.8. DEHP-DBP

In the DEHP-DBP combination, a clear reduction in cell viability (greater than 20%) was detected only when both compounds were applied simultaneously at the highest concentration analyzed (100 nM) (Figure 3B). In all other concentration combinations, changes in viability were minimal, indicating that cytotoxic effects only occurred under conditions of high combined exposure. This same condition also corresponded to the strongest synergistic interaction observed for this mixture. A slightly lower degree of synergy was observed for the combination of DBP at 10 nM with DEHP at 1 nM; however, this interaction was not associated with significant cytotoxicity, as cell viability was reduced by only about 5%. Consistent with this pattern, synergy analysis revealed weak but detectable interaction effects, with a mean synergy index of 0.33 ± 2.55 and a maximum synergistic area score of 1.98. While these values reflect moderate synergy, they indicate that interaction-dependent effects occur at specific concentration combinations, rather than being uniform across the entire dose range. In some cases, these interactions were associated with lower toxicity. The two-way ANOVA further emphasized the importance of the combined exposure, showing that the interaction between DEHP and DBP was the main factor influencing cell viability, explaining 59.08% of the total variance (*p* < 0.0001, F = 117.99, DFn = 4, DFd = 18). Analysis of the individual contributions revealed that DBP played a more prominent role than DEHP.

The main effect of DBP explained 25.38% of the variance and was highly significant (*p* < 0.0001, F = 101.38, DFn = 2, DFd = 18), while DEHP explained only a smaller, though still significant, proportion of the variance (13.29%; *p* < 0.0001, F = 53.10, DFn = 2, DFd = 18).

### 3.9. BADGE-DEHP

In the BADGE-DEHP mixture, a substantial decrease in cell viability (greater than 20%) was observed only when both compounds were administered together at the highest concentration tested (100 nM) (Figure 3C). At lower or mixed concentrations, cell viability remained virtually unchanged, suggesting that measurable cytotoxicity was only observed under conditions of maximum combined exposure. For this mixture, the combination of BADGE at 10 nM and DEHP at 1 nM elicited the strongest synergistic response observed among the tested conditions. In line with these observations, the synergy analysis indicated weak but measurable overall interaction effects, with a mean synergy index of 0.125 and a maximum synergistic area score of 1.79. While these values reflect limited synergy across the concentration range, they support the presence of interaction-dependent effects at specific dose combinations. The two-way ANOVA reinforced the predominance of combined effects, revealing that the interaction between BADGE and DEHP was the main driver of changes in cell viability, explaining 71.07% of the total variance (*p* < 0.0001, F = 48.89, DFn = 4, DFd = 18). The assessment of main effects showed that BADGE and DEHP contributed similarly to the overall response. BADGE alone explained 10.7% of the variance and was statistically significant (*p* = 0.0002, F = 14.73, DFn = 2, DFd = 18), while DEHP explained 11.68% of the variance (*p* < 0.0001, F = 16.08, DFn = 2, DFd = 18).

### 3.10. DEHP-PFOA

In the DEHP and PFOA mixture, a pronounced reduction in cell viability (greater than 20%) was observed only when both compounds were combined at the highest concentration analyzed (100 nM) (Figure 3D). Conversely, other combinations of concentrations of these pollutants produced minimal cytotoxic effects (generally less than 5%) and in some cases were associated with a slight increase in cell proliferation. Within this mixture, the combination of DEHP at 10 nM and PFOA at 1 nM produced the strongest synergistic response observed under the tested conditions. However, the DEHP and PFOA interactions were not uniformly synergistic across the concentration range. In fact, antagonistic effects were detected with multiple dose combinations, particularly when combining 10 nM PFOA with 100 nM DEHP. Consistent with this mixed interaction profile, the synergy analysis revealed an overall trend toward antagonism, reflected in a negative mean synergy index (−0.837 ± 2.47) and a low maximum synergistic area score (0.90). These findings indicate that while synergy emerged at specific high-dose combinations, antagonistic interactions predominated at intermediate concentrations. The two-way ANOVA highlighted the importance of the combined exposure, showing that the interaction between DEHP and PFOA was the main factor influencing cell viability, explaining 62.73% of the total variance (*p* < 0.0001, F = 54.60, DFn = 4, DFd = 18). Analysis of individual contributions revealed that PFOA had a stronger independent effect, explaining 19.14% of the variance (*p* < 0.0001, F = 33.31, DFn = 2, DFd = 18), while DEHP accounted for a smaller but still significant proportion (12.96%; *p* < 0.0001, F = 22.57, DFn = 2, DFd = 18).

### 3.11. Variability of Responses According to Mixture Concentrations

The mixtures showed statistically significant differences in cell viability when comparing specific concentration combinations. In several cases, modifying the concentration of one or both pollutants resulted in a clear change in the biological response compared to another combination. However, these changes did not follow a consistent or predictable trend across different mixtures or concentration ranges. In other words, the increases or decreases in cytotoxicity depended largely on the specific pair of compounds and their relative concentrations, rather than on a general dose-dependent pattern. Table 1 summarizes the statistical significance of these pairwise comparisons, highlighting which combinations differed significantly from one another. Taken together, these results underscore the complexity of mixture effects and indicate that the biological impact of combined exposures cannot be reliably inferred solely from concentration changes.

### 3.12. Cytotoxicity of BPA-DBP and BADGE-BPA Is Associated with Increased Intracellular ROS

**Figure 4:**
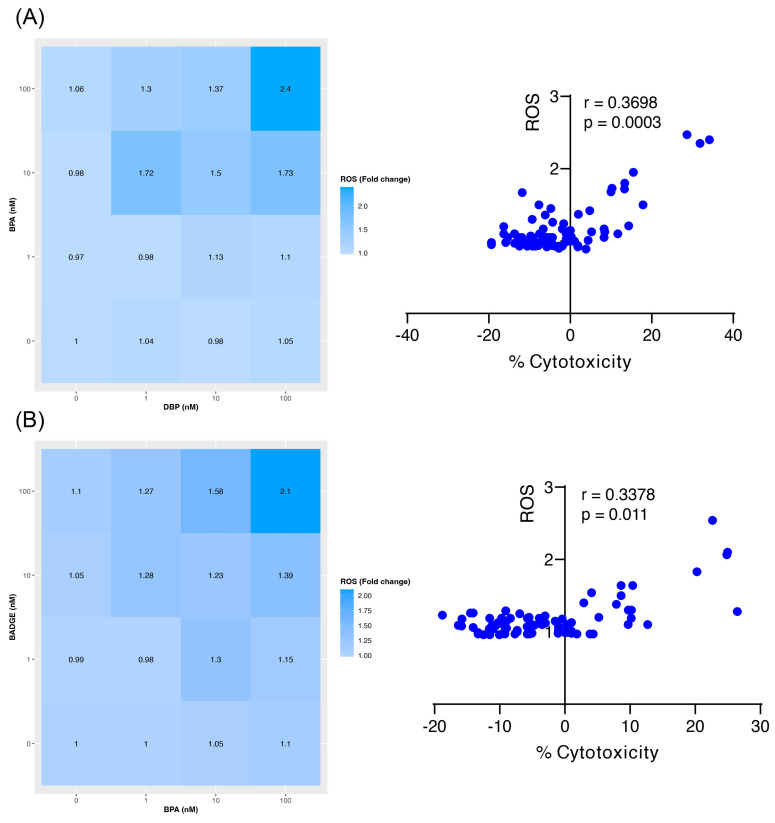
Induction of intracellular ROS and their association with cytotoxicity in BPA-DBP and BADGE-BPA combinations. The heatmaps on the left represent the change in intracellular ROS levels and the graphs on the right show their correlation with the cytotoxic effects for the pollutant combinations BPA-DBP (**A**) and BADGE-BPA (**B**). The panels on the left show the change in intracellular ROS levels, with color intensity indicating the magnitude of the response. The panels on the right present the corresponding correlation analyses between ROS levels and cytotoxicity. In the heatmaps, the first column represents the individual pollutant shown on the *Y*-axis, while the last row represents the individual pollutant shown on the *X*-axis. In both cases, these positions correspond to single-compound conditions at 0, 1, 10, and 100 nM.

Given that increased ROS production has been proposed as one of the main mechanisms underlying the synergistic effects of environmental pollutants [7], BPA–DBP and BADGE–BPA combinations (where synergy coincided with increased cytotoxicity) were evaluated to determine if these effects were associated with elevated levels of intracellular ROS. In the combination of BPA and DBP, intracellular ROS levels increased, with the most pronounced effects observed at the highest concentrations of both compounds (Figure 4A). Increased ROS production was also detected with BPA 10 nM combined with 1 nM and 100 nM DBP. Notably, a significant positive correlation was identified between the change in ROS folding and cytotoxicity (r = 0.3698, *p* = 0.0003), supporting an association between oxidative stress and the cytotoxic effects of this mixture. For the BADGE–BPA combination, intracellular ROS levels varied across the different concentration combinations tested (Figure 4B). The highest ROS levels were observed when both pollutants were tested at their maximum concentrations (100 nM), followed by the combination of BADGE at 100 nM and BPA at 10 nM. Similar to the BPA–DBP mixture, a significant positive correlation was detected between ROS fold change and cytotoxicity (r = 0.3378, *p* = 0.011), further supporting an association between oxidative stress and the cytotoxic effects of this combination.

## 4. Discussion

Despite growing concern about the health impacts of pollutant mixtures, research on synergistic interactions among environmental pollutants remains limited. A major challenge is the sheer number of possible pollutant combinations, each of which can interact in different ways. Furthermore, these interactions are often highly dose-dependent and can vary considerably depending on the experimental model, species, or biological system studied, making it difficult to compare or generalize the results. Taken together, these factors complicate both experimental design and data interpretation. In this context, the present study addresses a small but relevant part of this broader problem by providing new data on the interaction patterns of selected pollutants at environmentally relevant concentrations, thus contributing useful evidence to help improve our understanding of the toxicity of mixtures.

BPA and BADGE are commonly found in polycarbonate plastics and epoxy resins used in food and beverage containers, can linings, and other food contact materials, from which they can migrate into food and beverages. Meanwhile, DBP and DEHP are plasticizers widely used in flexible plastics, personal care products, medical devices, and household items, resulting in exposure through ingestion, inhalation, and skin contact. PFOA, a persistent member of the PFAS family, has been used in industrial processes and consumer products such as nonstick cookware, stain-resistant fabrics, and flame-retardant foams, and is frequently detected in contaminated water sources [14].

These compounds are associated with various adverse health effects: BPA and BADGE act as endocrine disruptors linked to reproductive, developmental, and metabolic alterations [15,16]; DBP and DEHP are primarily associated with reproductive toxicity, hormonal imbalance, and immunological and metabolic effects [17]; and PFOA exposure has been linked to immune dysfunction, liver and kidney toxicity, metabolic disorders, and an increased risk of cancer [18].

BPA has been detected in human serum at average concentrations of approximately 0.18 ng/mL (approximately 0.8 nM) in healthy adults, while individuals with greater exposure may have levels exceeding 3 ng/mL (approximately 13 nM) [19]. For BADGE-2H_2_O, reported serum concentrations in children range widely from 2.30 to 157.58 ng/mL (corresponding to approximately 7 to 460 nM) [20]. DBP has been measured at average serum levels of 0.05 µg/mL (approximately 180 nM), with peak concentrations reaching 3.42 µg/mL (approximately 12,300 nM). Similarly, mean DEHP concentrations of 0.05 µg/mL (approximately 130 nM) have been reported, with peak levels as high as 2.79 µg/mL (approximately 7150 nM) [21]. For PFOA, typical serum concentrations range from 0.1 to 30 ng/dL (equivalent to approximately 0.002 to 0.7 nM), with the highest values observed in highly exposed populations [22]. These reported concentrations highlight that human exposure commonly occurs within the nanomolar range, underscoring the importance of using low-dose experimental models to assess potential health effects.

In this study, concentrations of 1, 10, and 100 nM were applied uniformly to all pollutants to allow direct comparison of the binary mixture effects under controlled conditions. While this standardized design facilitates consistent assessment of interaction patterns, human exposure levels differ substantially among chemicals. Serum concentrations of DBP and DEHP can exceed 100 nM, whereas typical PFOA levels in the general population are usually below 1 nM, with higher values observed primarily in high-exposure groups. Therefore, the selected range represents a low-nanoscale experimental framework for assessing mixture interactions, rather than an exact replica of actual exposures to specific compounds.

Several of the pollutant combinations examined in this study have already been detected together in human biological samples, supporting their relevance to real-world exposures. BPA has frequently been detected alongside phthalates such as DBP and DEHP in urine and plasma, and these combined exposures have been associated with alterations in sex hormone levels, particularly during adolescence [23]. Phthalates have also been measured in conjunction with PFOA, and studies have reported links to altered thyroid hormone regulation in adolescent populations [24]. Furthermore, BPA and its derivative BADGE have been identified simultaneously in human urine samples, although clear health effects of this specific combination have not yet been established [25].

Since this study was based on reported plasma concentrations of the analyzed pollutants, a blood-derived cell model was selected to ensure its physiological relevance. Blood cells are directly exposed to circulating plasma levels and therefore represent a suitable in vitro system for evaluating the biological effects of these compounds [26]. Although the liver and kidney are among the main target organs for many environmental pollutants due to their role in the metabolism and excretion of xenobiotics [27,28,29], the availability of reliable quantitative human data on pollutant concentrations in these tissues is less consistent. Tissue measurements are usually derived from postmortem samples or surgical resections performed for clinical reasons, which can introduce variability related to disease status, treatment history, or postmortem redistribution [30]. For practical reasons, the NALM-6 leukemic cell line was used instead of primary healthy blood cells. Primary cells are often difficult to obtain in sufficient quantities, have limited proliferative capacity in vitro, and exhibit inter-donor variability that can affect reproducibility. In contrast, leukemic cell lines provide a stable, reproducible, and easily expandable model while maintaining relevance to circulating hematopoietic cells. NALM-6 cells are B-cell leukemia precursor cells isolated from the peripheral blood of a patient with acute lymphoblastic leukemia (ALL) [31], making them a practical and biologically relevant in vitro model.

Human myeloid leukemia cell lines express several cytochrome P450 (CYP) enzymes, including CYP1A1, CYP1B1, CYP2A6, CYP2A7, CYP2D6, and CYP2E1, whereas CYP2A13 and CYP2C9 are not detectable. The presence of the aryl hydrocarbon receptor (AhR) and its nuclear translocator (ARNT) indicates functional regulation of CYP1 family enzymes [32]. However, overall CYP expression remains lower than in primary hepatocytes, indicating limited metabolic capacity. Therefore, it is likely that the cytotoxic effects observed here in NALM-6 cells are driven predominantly by the parent compound rather than by metabolites of the pollutants.

The individual pollutants did not reduce cell viability at any of the concentrations tested. In most cases, they produced no effect or slightly increased MTT-derived viability values. In all binary mixtures, the most pronounced reductions in cell viability were consistently observed when both pollutants were combined at the highest concentrations analyzed (100 nM). These same conditions also produced the most pronounced synergistic responses. However, this correspondence was not consistently observed at other concentration combinations, where the relationship between synergy indices and absolute cytotoxic effects was lower. Indeed, the absolute reduction in cell viability observed for many combinations classified as “synergistic” was relatively modest, often less than 20% inhibition and, in some cases, less than 10%. Therefore, a statistically significant synergy score should be interpreted with caution when the underlying biological effect is small. While the calculated synergy value may be mathematically valid within the applied model, its toxicological relevance may be limited.

Notably, statistical analyses showed that, in all mixtures, the interaction between pollutants accounted for a greater proportion of the total variability in cell viability than the effects of the individual compounds alone. This indicates that the cellular response is largely driven by how the pollutants act together, rather than by their isolated toxicity. Among the compounds assessed, BADGE consistently exerted a greater influence on the mixture effects compared to the other pollutants. BPA and PFOA also contributed to interaction-driven responses, although their impact was generally less pronounced than that of BADGE.

The results of this study indicate that synergistic interactions among environmental pollutants may occur more frequently than previously recognized, although not all statistically identified interactions translate into clear biological relevance. In particular, the BPA-DBP combination was noteworthy, as it produced a high synergy score at the highest concentrations tested for both compounds, 100 nM, where the greatest degree of cytotoxicity was also observed (31%). Similarly, the BADGE-BPA mixture demonstrated one of the highest synergy scores and showed a corresponding increase in cytotoxicity, indicating a closer alignment between the interaction metric and the biological effect under those specific conditions. These findings highlighted the importance of distinguishing between statistical interaction and biologically significant responses, and of prioritizing mixtures where both parameters converge. Future studies should prioritize mixtures where statistical interaction metrics and measurable biological effects converge, as these cases are more likely to reflect toxicologically significant interactions.

The ROS overproduction is one of the mechanisms implicated in the harmful effects associated with synergistic interactions between pollutants [7]. Under physiological conditions, ROS are maintained at low and relatively stable levels, where they play essential roles in cell signaling and the regulation of cellular homeostasis [33]. To preserve this balance, cells rely on tightly regulated antioxidant defense systems, including enzymatic antioxidants and non-enzymatic scavengers [34]. However, these protective mechanisms can be overwhelmed under conditions of increased oxidative challenge, such as combined exposure to environmental pollutants [35]. When antioxidant defenses are insufficient, excess ROS can accumulate and react with proteins, lipids, carbohydrates, and nucleic acids, leading to structural damage, functional impairment, and, in severe cases, irreversible cell injury [34]. In this study, it was observed that combinations of BPA-DBP and BADGE-BPA significantly increase intracellular ROS levels. This increase in oxidative stress appears to contribute, at least in part, to the greater cytotoxicity and synergistic effects observed with these mixtures.

This study has several limitations that should be considered when interpreting the results. First, the experiments were performed using a single human cell line (NALM-6), which, while relevant for assessing hematological toxicity, cannot fully represent the complexity of responses in different tissues, cell types, or whole organisms. Consequently, the findings may not be directly extrapolated to in vivo conditions. Second, only binary mixtures were assessed, whereas real-world exposure typically involves more complex mixtures of multiple pollutants that can interact in nonlinear and unpredictable ways. Furthermore, the study focused on a limited concentration range and a single exposure duration, which may not reflect the time-dependent or chronic effects of low doses. Finally, the scope of the experimental design was limited by available financial resources. Budgetary constraints restricted the number of biological models, evaluation criteria, and mechanistic assays that could be included. Despite these limitations, the study provides valuable data on the toxicity of mixtures at environmentally relevant concentrations and highlights the need for further investigation.

## 5. Conclusions

This study demonstrates that biologically relevant interactions can occur between environmental pollutants at low nanomolar concentrations, consistent with reported human plasma levels. While not all statistically identified interactions resulted in toxicologically significant effects, specific combinations, particularly BPA-DBP and BADGE-BPA, produced increased cytotoxicity and synergistic responses under defined conditions. Increased ROS levels were partially associated with these effects. Overall, the findings emphasize the need to distinguish statistical synergy from toxicological relevance and indicate that mixture effects cannot always be predicted from individual compound assessments. Consequently, further research is needed to better characterize the mechanisms, dose dependencies, and long-term effects of pollutant mixtures on different biological systems. Expanding this knowledge base will be essential for refining environmental regulations and developing more protective frameworks that consider the combined effects of chemical exposures.

## Figures and Tables

**Table 1:** Multiple comparisons of concentration combinations in binary mixtures. Statistical differences were assessed using two-way ANOVA, followed by Tukey’s post hoc test for multiple comparisons. Significance levels are indicated as follows: NS, not significant (*p* > 0.05); * *p* ≤ 0.05; ** *p* ≤ 0.01; *** *p* ≤ 0.001; **** *p* ≤ 0.0001.

Comparison	BADGE- BPA	BADGE- DBP	DEHP- BPA	DBP- BPA	PFOA- BPA	BADGE- PFOA	PFOA- DBP	DEHP- DBP	BADGE- DEHP	DEHP- PFOA
1 nM:1 nM vs. 1 nM:10 nM	*	*	****	****	**	**	*	****	**	***
1 nM:1 nM vs. 1 nM:100 nM	NS	NS	***	NS	*	NS	NS	NS	NS	NS
1 nM:1 nM vs. 10 nM:1 nM	***	NS	****	****	**	*	****	*	****	****
1 nM:1 nM vs. 10 nM:10 nM	NS	NS	*	NS	NS	NS	NS	**	NS	NS
1 nM:1 nM vs. 10 nM:100 nM	NS	NS	***	*	NS	NS	***	*	NS	*
1 nM:1 nM vs. 100 nM:1 nM	NS	NS	*	NS	NS	NS	NS	NS	NS	NS
1 nM:1 nM vs. 100 nM:10 nM	NS	NS	****	****	**	*	***	****	NS	**
1 nM:1 nM vs. 100 nM:100 nM	****	****	****	****	****	****	****	****	****	****
1 nM:10 nM vs. 1 nM:100 nM	***	**	**	****	NS	*	***	****	NS	*
1 nM:10 nM vs. 10 nM:1 nM	NS	NS	**	NS	NS	NS	***	*	NS	NS
1 nM:10 nM vs. 10 nM:10 nM	*	**	****	****	**	NS	NS	NS	*	*
1 nM:10 nM vs. 10 nM:100 nM	NS	NS	***	**	NS	NS	NS	*	NS	NS
1 nM:10 nM vs. 100 nM:1 nM	***	**	****	****	NS	NS	*	****	*	***
1 nM:10 nM vs. 100 nM:10 nM	NS	NS	NS	NS	NS	NS	NS	NS	NS	NS
1 nM:10 nM vs. 100 nM:100 nM	*	*	NS	***	NS	*	****	****	****	****
1 nM:100 nM vs. 10 nM:1 nM	****	**	NS	****	NS	NS	****	**	**	**
1 nM:100 nM vs. 10 nM:10 nM	NS	NS	NS	NS	*	NS	**	**	NS	NS
1 nM:100 nM vs. 10 nM:100 nM	**	NS	NS	NS	NS	NS	****	**	NS	NS
1 nM:100 nM vs. 100 nM:1 nM	NS	NS	NS	NS	NS	NS	NS	NS	NS	NS
1 nM:100 nM vs. 100 nM:10 nM	**	*	NS	***	NS	NS	****	****	NS	NS
1 nM:100 nM vs. 100 nM:100 nM	****	****	****	****	*	****	****	****	****	****
10 nM:1 nM vs. 10 nM:10 nM	****	**	NS	****	*	NS	***	NS	***	**
10 nM:1 nM vs. 10 nM:100 nM	NS	NS	NS	*	NS	NS	NS	NS	***	NS
10 nM:1 nM vs. 100 nM:1 nM	****	**	NS	****	NS	NS	****	****	***	****
10 nM:1 nM vs. 100 nM:10 nM	NS	NS	NS	NS	NS	NS	*	NS	**	NS
10 nM:1 nM vs. 100 nM:100 nM	NS	*	****	****	*	**	NS	****	***	****
10 nM:10 nM vs. 10 nM:100 nM	NS	NS	NS	*	NS	NS	NS	NS	NS	NS
10 nM:10 nM vs. 100 nM:1 nM	NS	NS	NS	NS	NS	NS	NS	****	NS	NS
10 nM:10 nM vs. 100 nM:10 nM	NS	**	**	****	**	NS	NS	NS	NS	NS
10 nM:10 nM vs. 100 nM:100 nM	****	****	****	****	****	****	****	****	****	****
10 nM:100 nM vs. 100 nM:10 nM	**	NS	NS	*	NS	NS	***	****	NS	*
10 nM:100 nM vs. 100 nM:1 nM	NS	NS	NS	NS	NS	NS	NS	NS	NS	NS
10 nM:100 nM vs. 100 nM:100 nM	**	***	****	****	****	***	**	****	****	****
100 nM:1 nM vs. 100 nM:10 nM	**	**	**	****	NS	NS	***	****	NS	**
100 nM:1 nM vs. 100 nM:100 nM	****	****	****	****	***	***	****	****	****	****
100 nM:10 nM vs. 100 nM:100 nM	**	**	**	****	NS	**	***	****	****	****

## Data Availability

The original contributions presented in this study are included in the article. Further inquiries can be directed to the corresponding author.

## Abbreviations

The following abbreviations are used in this manuscript:

AhR	Aryl hydrocarbon receptor
ALL	Acute lymphoblastic leukemia
ANOVA	Analysis of variance
BPA	Bisphenol A
BADGE	Bisphenol A diglycidyl ether
CYP	Cytochrome P450
DBP	Dibutyl phthalate
DEHP	Di(2-ethylhexyl) phthalate
DCFH-DA	2′,7′-dichlorodihydrofluorescein diacetate
DMSO	Dimethyl sulfoxide
MTT	3-(4,5-dimethylthiazol-2-yl)-2,5-diphenyltetrazolium bromide
PFOA	Perfluorooctanoic acid
ROS	Reactive oxygen species

## References

[1] Lagunas-Rangel F.A., Kudłak B., Liu W., Williams M.J., Schiöth H.B. (2022). The Potential Interaction of Environmental Pollutants and Circadian Rhythm Regulations That May Cause Leukemia. Crit. Rev. Environ. Sci. Technol..

[2] Lagunas-Rangel F.A. (2026). Pollutants and Cancer Therapy. Environ. Res..

[3] Landrigan P.J., Fuller R., Acosta N.J.R., Adeyi O., Arnold R., Basu (Nil) N., Baldé A.B., Bertollini R., Bose-O’Reilly S., Boufford J.I. (2018). The Lancet Commission on Pollution and Health. Lancet.

[4] Fuller R., Landrigan P.J., Balakrishnan K., Bathan G., Bose-O’Reilly S., Brauer M., Caravanos J., Chiles T., Cohen A., Corra L. (2022). Pollution and Health: A Progress Update. Lancet Planet. Health.

[5] Bopp S.K., Barouki R., Brack W., Dalla Costa S., Dorne J.-L.C.M., Drakvik P.E., Faust M., Karjalainen T.K., Kephalopoulos S., van Klaveren J. (2018). Current EU Research Activities on Combined Exposure to Multiple Chemicals. Environ. Int..

[6] Goodson W.H., Lowe L., Carpenter D.O., Gilbertson M., Manaf Ali A., Lopez de Cerain Salsamendi A., Lasfar A., Carnero A., Azqueta A., Amedei A. (2015). Assessing the Carcinogenic Potential of Low-Dose Exposures to Chemical Mixtures in the Environment: The Challenge Ahead. Carcinogenesis.

[7] Lagunas-Rangel F.A., Linnea-Niemi J.V., Kudłak B., Williams M.J., Jönsson J., Schiöth H.B. (2022). Role of the Synergistic Interactions of Environmental Pollutants in the Development of Cancer. Geohealth.

[8] Geary N. (2013). Understanding Synergy. Am. J. Physiol.-Endocrinol. Metab..

[9] Niu J., Straubinger R.M., Mager D.E. (2019). Pharmacodynamic Drug–Drug Interactions. Clin. Pharmacol. Ther..

[10] Jatkowska N., Kudłak B., Lewandowska P., Liu W., Williams M.J., Schiöth H.B. (2021). Identification of Synergistic and Antagonistic Actions of Environmental Pollutants: Bisphenols A, S and F in the Presence of DEP, DBP, BADGE and BADGE·2HCl in Three Component Mixtures. Sci. Total Environ..

[11] Cedergreen N. (2014). Quantifying Synergy: A Systematic Review of Mixture Toxicity Studies within Environmental Toxicology. PLoS ONE.

[12] Brack W. (2015). The Challenge: Prioritization of Emerging Pollutants. Environ. Toxicol. Chem..

[13] Ianevski A., Giri A.K., Aittokallio T. (2022). SynergyFinder 3.0: An Interactive Analysis and Consensus Interpretation of Multi-Drug Synergies across Multiple Samples. Nucleic Acids Res..

[14] Lagunas-Rangel F.A., Åberg M., Liao S., Espinelli-Amorim F., Tummaramatti-Hanumant R., Jackeviča L., Briviba M., Liu W., Fredriksson R., Alsehli A.M. (2025). Extensive Analysis of Pollutant Interactions: Identification of Deleterious Synergistic Effects at Environmentally Relevant Dose Levels Using Drosophila and Mammalian Cells. Sci. Total Environ..

[15] Poole A., van Herwijnen P., Weideli H., Thomas M.C., Ransbotyn G., Vance C. (2004). Review of the Toxicology, Human Exposure and Safety Assessment for Bisphenol A Diglycidylether (BADGE). Food Addit. Contam..

[16] vom Saal F.S., Nagel S.C., Coe B.L., Angle B.M., Taylor J.A. (2012). The Estrogenic Endocrine Disrupting Chemical Bisphenol A (BPA) and Obesity. Mol. Cell. Endocrinol..

[17] Heudorf U., Mersch-Sundermann V., Angerer J. (2007). Phthalates: Toxicology and Exposure. Int. J. Hyg. Environ. Health.

[18] Li K., Gao P., Xiang P., Zhang X., Cui X., Ma L.Q. (2017). Molecular Mechanisms of PFOA-Induced Toxicity in Animals and Humans: Implications for Health Risks. Environ. Int..

[19] Mantzouki C., Bliatka D., Iliadou P.K., Margeli A., Papassotiriou I., Mastorakos G., Kousta E., Goulis D.G. (2019). Serum Bisphenol A Concentrations in Men with Idiopathic Infertility. Food Chem. Toxicol..

[20] Kuwamura M., Tanaka K., Onoda A., Taki K., Koriyama C., Kitagawa K., Kawamoto T., Tsuji M. (2024). Measurement of Bisphenol A Diglycidyl Ether (BADGE), BADGE Derivatives, and Bisphenol F Diglycidyl Ether (BFDGE) in Japanese Infants with NICU Hospitalization History. BMC Pediatr..

[21] Li S., Dai J., Zhang L., Zhang J., Zhang Z., Chen B. (2011). An Association of Elevated Serum Prolactin with Phthalate Exposure in Adult Men. Biomed. Environ. Sci..

[22] Niu Z., Duan Z., He W., Chen T., Tang H., Du S., Sun J., Chen H., Hu Y., Iijima Y. (2024). Kidney Function Decline Mediates the Adverse Effects of Per- and Poly-Fluoroalkyl Substances (PFAS) on Uric Acid Levels and Hyperuricemia Risk. J. Hazard. Mater..

[23] Vu Huynh Q.T., Ban H.T., Vuong N.L., Khanh N.P. (2024). The Relationship between Bisphenol A and Phthalates with Precocious Puberty in Vietnamese Children. J. Pediatr. Endocrinol. Metab..

[24] Rodríguez-Carrillo A., Salamanca-Fernández E., den Hond E., Verheyen V.J., Fábelová L., Murinova L.P., Pedraza-Díaz S., Castaño A., García-Lario J.V., Remy S. (2023). Association of Exposure to Perfluoroalkyl Substances (PFAS) and Phthalates with Thyroid Hormones in Adolescents from HBM4EU Aligned Studies. Environ. Res..

[25] Liu M., Jia S., Dong T., Han Y., Xue J., Wanjaya E.R., Fang M. (2019). The Occurrence of Bisphenol Plasticizers in Paired Dust and Urine Samples and Its Association with Oxidative Stress. Chemosphere.

[26] Krewski D., Acosta D., Andersen M., Anderson H., Bailar J.C., Boekelheide K., Brent R., Charnley G., Cheung V.G., Green S. (2010). Toxicity Testing in the 21st Century: A Vision and a Strategy. J. Toxicol. Environ. Health Part B.

[27] Thayer K.A., Doerge D.R., Hunt D., Schurman S.H., Twaddle N.C., Churchwell M.I., Garantziotis S., Kissling G.E., Easterling M.R., Bucher J.R. (2015). Pharmacokinetics of Bisphenol A in Humans Following a Single Oral Administration. Environ. Int..

[28] Chang L.-W., Hou M.-L., Tsai T.-H. (2013). Pharmacokinetics of Dibutyl Phthalate (DBP) in the Rat Determined by UPLC-MS/MS. Int. J. Mol. Sci..

[29] Loccisano A.E., Campbell J.L., Andersen M.E., Clewell H.J. (2011). Evaluation and Prediction of Pharmacokinetics of PFOA and PFOS in the Monkey and Human Using a PBPK Model. Regul. Toxicol. Pharmacol..

[30] Pounder D.J., Jones G.R. (1990). Post-Mortem Drug Redistribution—A Toxicological Nightmare. Forensic Sci. Int..

[31] Hurwitz R., Hozier J., Lebien T., Minowada J., Gajl-Peczalska K., Kubonishi I., Kersey J. (1979). Characterization of a Leukemic Cell Line of the Pre-B Phenotype. Int. J. Cancer.

[32] Nagai F., Hiyoshi Y., Sugimachi K., Tamura H. (2002). Cytochrome P450 (CYP) Expression in Human Myeloblastic and Lymphoid Cell Lines. Biol. Pharm. Bull..

[33] Kirtonia A., Sethi G., Garg M. (2020). The Multifaceted Role of Reactive Oxygen Species in Tumorigenesis. Cell. Mol. Life Sci..

[34] Brieger K., Schiavone S., Miller F.J., Krause K. (2012). Reactive Oxygen Species: From Health to Disease. Swiss Med. Wkly..

[35] Kamp D.W., Greenberger M.J., Sbalchierro J.S., Preusen S.E., Weitzman S.A. (1998). Cigarette Smoke Augments Asbestos-Induced Alveolar Epithelial Cell Injury: Role of Free Radicals. Free Radic. Biol. Med..

